# Elucidating the identity of resistance mechanisms to prednisolone exposure in acute lymphoblastic leukemia cells through transcriptomic analysis: A computational approach

**DOI:** 10.1186/2043-9113-1-36

**Published:** 2011-12-20

**Authors:** Emmanouil G Sifakis, George I Lambrou, Andriana Prentza, Spiros Vlahopoulos, Dimitris Koutsouris, Fotini Tzortzatou-Stathopoulou, Aristotelis A Chatziioannou

**Affiliations:** 1 School of Electrical and Computer Engineering, National Technical University of Athens, Athens, Greece; 2 First Department of Pediatrics, University of Athens, Choremeio Research Laboratory, Athens, Greece; 3 Department of Digital Systems, University of Piraeus, Piraeus, Greece; 4 University Research Institute for Genetic and Malignant Diseases of Childhood, University of Athens, Athens, Greece; 5 Institute of Biological Research & Biotechnology, National Hellenic Research Foundation, Athens, Greece

**Keywords:** acute lymphoblastic leukemia, DNA microarray analysis, gene ontology, glucocorticoid resistance, intrinsic vs. acquired

## Abstract

**Background:**

It has been shown previously that glucocorticoids exert a dual mechanism of action, entailing cytotoxic, mitogenic as well as cell proliferative and anti-apoptotic responses, in a dose-dependent manner on CCRF-CEM cells at 72 h. Early gene expression response implies a dose-dependent dual mechanism of action of prednisolone too, something reflected on cell state upon 72 h of treatment.

**Methods:**

In this work, a generic, computational microarray data analysis framework is proposed, in order to examine the hypothesis, whether CCRF-CEM cells exhibit an intrinsic or acquired mechanism of resistance and investigate the molecular imprint of this, upon prednisolone treatment. The experimental design enables the examination of both the dose (0 nM, 10 nM, 22 uM, 700 uM) effect of glucocorticoid exposure and the dynamics (early and late, namely 4 h, 72 h) of the molecular response of the cells at the transcriptomic layer.

**Results:**

In this work, we demonstrated that CCRF-CEM cells may attain a mixed mechanism of response to glucocorticoids, however, with a clear preference towards an intrinsic mechanism of resistance. Specifically, at 4 h, prednisolone appeared to down-regulate apoptotic genes. Also, low and high prednisolone concentrations up-regulates genes related to metabolism and signal-transduction in both time points, thus favoring cell proliferative actions. In addition, regulation of NF-κB-related genes implies an inherent mechanism of resistance through the established link of NF-κB inflammatory role and GC-induced resistance. The analysis framework applied here highlights prednisolone-activated regulatory mechanisms through identification of early responding sets of genes. On the other hand, study of the prolonged exposure to glucocorticoids (72 h exposure) highlights the effect of homeostatic feedback mechanisms of the treated cells.

**Conclusions:**

Overall, it appears that CCRF-CEM cells in this study exhibit a diversified, combined pattern of intrinsic and acquired resistance to prednisolone, with a tendency towards inherent resistant characteristics, through activation of different molecular courses of action.

## Background

Resistance to glucocorticoids (GC) is considered to be one of the most important factors in the prognosis of leukemia [1,2]. In a previous study, it has been shown that when a resistant T-cell leukemia cell line (CCRF-CEM) is treated with prednisolone, the drug exerts a dual (biphasic) effect on these cells [3]. At low doses, prednisolone has a mitogenic/anti-apoptotic effect, whereas at higher doses it manifests a cytotoxic/mitogenic effect. Also, it has been shown that the actual underlying effect of prednisolone, either mitogenic or cytotoxic, becomes apparent at 72 h of prednisolone exposure, providing evidence for activation of a cellular, homeostatic, feedback mechanism at the transcriptional or translational layer (protein synthesis) [3]. 

In addition, it remains elusive whether cells possess inherent mechanisms inducing GC tolerance on them, or their responce upon GC treatment is one of gradual adjustment, meaning that originally sensitive cells become resistant. Thus, as glucocorticoid receptor regulates directly or indirectly several thousands of genes, this partly refers to activation of genes related to anti-apoptosis and mitogenesis. In this sense, those mechanisms may possibly, through intricate, regulatory actions and cross-talks, confer to the induction of resistance in leukemic cells. Apoptosis evasion, or proliferation stimulation are two alternative mechanisms through which cells exhibit resistance. In the present work we refer to acute lymphoblastic leukemia (ALL), though glucocorticoid treatment belongs to the first-line of medications against lymphoid malignancies in general [4,5]. There is adequate evidence supporting a far more intricate mechanism of resistance to glucocorticoids than mere down-regulation of steroid receptors. 

In this sense, several, possible resistance mechanisms of leukemic cells to glucocorticoid administration have been proposed, like the presence of somatic mutations on the GR gene that may lead to aberrant regulation of the receptor through intracellular signaling. Besides, several polymorphisms, but not somatic mutations, have been found in normal and ALL populations, not linked to resistance or sensitivity induction though, either *in vivo *or *in vitro*[6,7]. Other GC resistance scenarios are emphasizing in defects in intracellular signaling pathways that involve interactions of GR with other sequence-specific transcription factors, such as AP-1 and Nuclear Factor kappa-light-chain-enhancer of activated B cells (NF-κB) [8]. In a normal cell, ligand-activated GR may potentially interfere with transcription factor c-Jun or p65 NF-κB and thereby repress genes promoting cell proliferation and cell survival [6,9,10]. GR-dependent inhibition of the transcription factor p65 NF-κB, plays a significant role in the manifestation of apoptotic and anti-apoptotic effects of GR in leukemia cells and has been identified as a pivotal component of the mechanism of cancer cell resistance to chemotherapy [9]. Previous studies of GC effects on leukemia cells identified *c-myc *and cyclin D3 as early GR-regulated targets, in GC-sensitive cells [11]. Further studies showed that introduction of a conditionally expressed cyclin-dependent kinase inhibitor p16 (INK4A) gene, sensitized GC-resistant leukemia cells, through induction of cell cycle arrest [12].

Thus, p16 inactivation may change GR levels, affecting GR-mediated gene regulation and resulting in resistance to GCs. For this purpose, the parental CCRF-CEM cell line was chosen as the system of study for the effects of prednisolone treatment, a T-cell leukemia cell line characterized by a mutation (L753F) on one GR gene allele that impairs ligand binding [13]. It is known that both the DNA and ligand binding domains of the GR are required in order to repress NF-κB transactivation [14]. Interestingly, concerning the question whether this mutation would affect GC resistance, it has been reported previously that both the GC-resistant, as well as the GC-sensitive CCRF-CEM subclones, express heterogeneous populations of the GR (GR^wt^/GRL753F) [15,16]. The CCRF-CEM cell line has been reported to be resistant to GCs, presumably due to the accumulation of more resistant variants after long periods of prolonged culture [17]. In addition, utilization of an *in vitro *system provides reproducibility, an expedient system to systematically examine the impact of intracellular signals and at the same time minimize the effect of undesired crosstalks introduced by other *in vivo-*participating systems. 

A detailed molecular explanation of the intricate mechanisms, underlying the resistance phenotype to GC-induced apoptosis, remains elusive. The present work proposes a rational computational framework in order to aid the elucidation of the question whether the system under study, has intrinsic or acquired mechanisms of resistance. Our presumption is that the system in study possesses an intrinsic mechanism of resistance to glucocorticoids i.e. prednisolone. Using the proposed computational analysis workflow, we have analyzed microarray data from two time points (4 and 72 h treatment) and three different concentrations (10 nM, 22 uM and 700 uM). For the 4 h time point, we used a 1.2 k platform, comprising of cancer specific genes, which has been reported and analyzed previously [3,18]. In order to expand our view of prednisolone effects on the cell line, we used a 4.8 k platform. Genes included in the 1.2 k platform are also represented in the 4.8 k platform. Data analysis was performed in order to find groups of genes associated with characteristics related to anti-apoptosis and apoptosis, cell cycle arrest, drug resistance etc.

## Methods

### Data collection

The CCRF-CEM cell line was obtained from the European Collection of Cell Cultures (ECACC). Concentrations of prednisolone (Pharmacia) were: 0 uM (control), 10 nM, 22 uM, and 700 uM. In general, all three prednisolone concentrations correspond to *in vivo *dosages administrated intravenously to children at ages between 1 month and 12 years old [3]. Specifically, the 10 nM and 700 uM prednisolone concentrations were chosen as indicative of manifestation of specific phenotypic effects, i.e. anti-apoptosis accompanied with mitogenic effect and cytotoxicity accompanied with resistance, as observed by flow cytometry [3]. Moreover, the high concentration (700 uM) used, is similar to concentrations used in different studies in CCRF-CEM cells [19] as well as primary cell cultures derived from childhood ALL patients [20]. The 22 uM prednisolone concentration was chosen as an intermediate concentration between the aforementioned two.

RNA was isolated with Trizol (Invitrogen Inc.) according to the manufacturer's instructions. At least 40 ug of RNA from each sample was used. cDNA microarray chips from two platforms (1.2 k and 4.8 k) were obtained from TAKARA (IntelliGene^® ^Human Cancer CHIP Version 4.0 and IntelliGene^® ^II CHIP, respectively). Hybridization was performed with the CyScribe Post-Labeling kit (RPN5660, Amersham) as described by the manufacturer [3]. The experimental setups consisted of the five following pairs: control vs. 10 nM prednisolone at 4 h (designated as '1'), 10 nM vs. 700 uM prednisolone at 4 h (designated as '2'), control vs. 700 uM prednisolone at 4 h (designated as '3'), 22 uM vs. 700 uM prednisolone at 72 h (designated as '4'), and control vs. 700 uM prednisolone at 72 h (designated as '5'). In this study, triplicate hybridizations are utilized for the 4 h experiments. Experimental pairs were co-hybridized on the same slide, each stained with a different fluorophore. Fluorophores used were Cy3 and Cy5. Slides were scanned with the ScanArray 4000XL microarray scanner. Images were generated with ScanArray microarray acquisition software (GSI Lumonics, USA). Image analysis was performed with the ImaGene^® ^6.0 software (Biodiscovery Inc., USA). The raw datasets have been deposited in NCBI's Gene Expression Omnibus (GEO), and are accessible through GEO Series accession number [GEO: GSE28154].

### Data preprocessing

A common for both platforms data preprocessing stage, namely the median intensity value in each channel, was applied to the raw data. Specifically, the well performing robust version of the robust loess-based background correction (rLsBC) approach, as proposed by [21], was applied. rLsBC assumes that the background noise affects the spot intensities in a multiplicative manner [22]. Instead of using the measurements of the local (feature-related) background for the correction, rLsBC utilizes the regression estimate of the logarithmic background distribution *B*^*R, G *^according to the logarithmic foreground intensity *F*^*R, G *^for each channel (*R*: Red and *G*: Green). Thus, rLsBC provides a robust estimation of the channel-specific background noise, utilized to background-correct the logarithmic foreground intensities:

$$F_{c}^{R,G}=F^{R,G}-B_{l}^{R,G}$$

where $F_{c}^{R,G}$ is the logarithmic background-corrected foreground intensity, and $B_{l}^{R,G}$ the robust estimate of background noise, for each channel. The absolute background-corrected foreground intensity $f_{c}^{R,G}$ for each channel is then calculated as:

$$f_{c}^{R,G}=2^{F_{c}^{R,G}}$$

In order to reduce the complexity of the data set, we followed the replicate averaging approach proposed by [23]. In this approach, instead of estimating a constant *c *and utilizing it to adjust each of the individual replicate measurements, equivalently the replicates were averaged by taking their geometric mean, that is:

$$f_{a}^{R,G}=\sqrt[{3}]{f_{r_{1}}^{R,G}\cdot f_{r_{2}}^{R,G}\cdot f_{r_{3}}^{R,G}},$$

where $f_{r_{i}}^{R,G}$ is the (background-corrected) foreground intensity of the replicate *r_i_*, *i *= 1, 2, 3, and $f_{a}^{R,G}$ is the averaged foreground intensity across all replicates (henceforth referred simply as signal intensity), for each channel (Red and Green). 

Since outliers can significantly influence one or more of the subsequent processing steps, extreme outlier values, that is signal intensities deviating more than 3 interquartile distances from the first or the third quartile [24], were identified and excluded in an iterative process.

The signal intensities of each dataset were further normalized in order to mitigate the effect of extraneous, non-biological variation in the measured gene expression levels. The robust version of the intensity-dependent scatter-plot smoother loess [25,26] with a quadratic polynomial model was applied to the M-A scatter-plot [27], where *M *and *A *are the log-ratio and log-mean, respectively:

$$\begin{array}{ccc} M & =F^{R}-F^{G} & \\ A & =\frac{F^{R}+F^{G}}{2} \end{array}$$

where *F^R ^*and *F^G ^*are the corresponding logarithmic signal intensities for each channel. The smoothing parameter of the loess procedure used equals to 10%, which was considered appropriate for the relatively small number of probes attached in the microarrays.

In order to identify and remove dubious features, a filtering approach was followed based on (i) the loop-design [28-31] used for the experimental setups at 4 h (experiments '1', '2', and '3'), and (ii) the philosophy of the replicate filtering approach proposed by [32]. In particular, the fold changes of experiment '1' (control vs. 10 nM prednisolone) and experiment '2' (10 nM vs. 700 uM prednisolone) should roughly equal the fold change in experiment '3' (control vs. 700 uM prednisolone), that is:

$$\frac{f_{1}^{R}}{f_{1}^{G}}\cdot \frac{f_{2}^{R}}{f_{2}^{G}}=\frac{f_{3}^{R}}{f_{3}^{G}}$$

where $f_{i}^{R,G}$ are the signal intensity of the experiment *i*, *i *= 1, 2, 3, for each channel, or equivalently, the following quantity should ideally be equal to zero:

$$\begin{array}{ccc} LdF & =\log_{2}\left(\frac{\frac{f_{1}^{R}}{f_{1}^{G}}\cdot \frac{f_{2}^{R}}{f_{2}^{G}}}{\frac{f_{3}^{R}}{f_{3}^{G}}}\right)= & \\ & =M_{1}+M_{2}-M_{3} \end{array}$$

where *M_i _*is the log-ratio of the experiment *i*, *i *= 1, 2, 3. Thus, we sought to filter out features whose *LdF *deviates greatly from this expected value of zero. We calculated the mean and standard deviation (SD) of *LdF*, and eliminated features whose *LdF *is greater than 2 SD from the mean. The selected number of standard deviations of the mean ensures that on the one hand the features used for the subsequent analysis roughly comply with this loop-design rule with relatively high confidence, while on the other hand selection thresholds are not too stringent, and thus reject potentially interesting genes.

### Data integration

Before proceeding to the cross-platform analysis, since two different microarray platforms (IntelliGene^® ^Human Cancer CHIP Version 4.0 and IntelliGene^® ^II CHIP) were utilized in the present study, we chose to perform data integration at a lower level [33], instead of conducting a meta-analysis. That is, the preprocessed datasets of each array were combined to a single, unified dataset, in which standard statistical procedures were applied. Nonetheless, whenever applicable to the nature of the present study, some of the key issues that need to be addressed, i.e. preprocessing, preparation and annotation of the individual datasets, complied to the guidelines suggested by [34].

In order to perform data integration, two main issues had to be resolved: (i) matching reporters on the two microarray platforms, and (ii) normalizing data to address platform related differences [35].

The first task was rather straightforward, since both platforms were obtained from the same manufacturer and many reporters were common between the 1.2 k and the 4.8 k platforms. Specifically, each reporter-level identifier (GenBank accession numbers) was mapped to a UniGene identifier (UniGene Cluster ID) [36-40]. The mapping was performed through the web-based tool SOURCE [41] in the 19th of November 2010, simultaneously for both platforms, in order to avoid inconsistencies [42]. All mapped reporter-level identifiers had one-to-one relationship with the gene-level identifiers that is, each reporter was associated with a single UniGene identifier and no more than one reporter was mapped to the same UniGene identifier. Reporters having insufficient information to be mapped to any gene-level identifier were omitted. Thus, a fully updated set of unique gene-level identifiers was generated for each platform. The intersection of these two sets formed a common gene set (CGS) consisting of 490 UniGene identifiers common in all setups, which was utilized for the subsequent analysis.

Then, a cross-platform normalization procedure was applied in order to address batch effect issues, namely the median rank score (MRS) normalization method [43,44]. After choosing a microarray set as reference, this simple approach replaces the expression values for all the others (non-reference) microarray sets by median expression values of genes from the reference set. The reference set of choice was the 4.8 k platform set, since it outperforms in data quality when compared to the 1.2 k one [43]. The improved comparability of the data from the two different platforms after cross-platform normalization is shown in Additional File 1, where it can be seen that the distributions of gene expression values derived from the 1.2 k and 4.8 k platforms are more similar after application of MRS in comparison to non-integrated data. 

The following analysis steps were performed on the integrated gene expression values of the CGS.

### Identification of differentially expressed genes

In order to identify potential, differentially expressed (DE) genes for each slide, whenever the experimental design did not include replicates for the same condition, we adjusted a selection process, exploiting the intensity-dependent calculation of the standard Z-score [32] of each feature within each slide. This approach utilizes a sliding window to calculate smoothed local means and standard deviations (SDs), which are then used to calculate the Z-score of the logarithmic ratio values for each data point in the normalized MA-plot. At the present study, a sliding window of width equal to 20% of the total number of data points was utilized. The selected percentage on one hand represents a plausible tradeoff between the adequate sample size for statistics calculation, and neighbourhood sensitivity regarding the intensity-dependent action. The DE genes per experiment were identified at a confidence level of 95%.

### Cluster analysis

In order to partition the gene expression profiles throughout all five experimental setups and exploit similarities in the expression profiles, for surmising functional relevance, possibly through common regulatory actions, which orchestrate genomic expression with respect to the studied phenotypes in this study, an unsupervised cluster analysis was utilized.

The unsupervised clustering was applied to an appropriately selected subset of the CGS, since simulations have indicated that keeping irrelevant genes during cluster analysis, results in reduced accuracy [45]. Specifically, each gene of the CGS was ranked by SD across experiments. The top 100 genes with the highest SD (SD100) were selected for downstream analysis. As demonstrated in the recent thorough evaluation study for two-channel microarray data [46], this widely used method is one of the best performing gene selection approaches.

The method utilized for cluster analysis was the k-means clustering [47-49], considered as one of the best performing clustering options in particular for microarray class discovery studies [46]. The k-means algorithm applied [50] uses the squared Euclidean as a distance measure, which has been frequently found to outperform, regargding ratio-based measurements [51]. Also, in order to evade the problem of possible local minima, the algorithm is repeated a number of executions with different initializations. Specifically, the clustering procedure is repeated 100 times, each with a new set of initial cluster centroid positions (seeds), selected at random, and the best execution (the one that minimizes the sum, over all clusters, of the within-cluster sums of object-to-cluster-centroid distances) is taken as the final result.

In order to predict the optimal number of clusters for interpretation, which constitutes a fundamental problem in unsupervised clustering, a cluster validity measure was applied. The validity measure of choice was the average silhouette width for the entire dataset *S *comprising *N *objects [52,53]:

$${\bar{S}}_{k}=\frac{1}{N}\underset{i\in S}{\sum }S_{i}$$

where *S_i _*is the silhouette width of object *i*, which is defined as:

$$S_{i}=\frac{b_{i}-a_{i}}{\max\left(a_{i},b_{i}\right)}$$

where *a_i _*is the average distance between object *i *and all the other objects in the cluster, and *b_i _*is the minimum of the average distances between *i *and objects in other clusters. To determine the optimal number of clusters [54], the clustering was executed for *k *varying between 2 to 30. For each *k*, the aforementioned *k*-means algorithm was repeated 1,000 times. The best (maximum) values of ${\bar{S}}_{k}$ obtained by each *k *were plotted as the function of *k *(Additional File 2). As illustrated in this figure, since the plot did not exhibit any specific increasing or decreasing trend, we sought for its maximum value [54]. Thus, the optimal cluster number was the one corresponding to the maximum value of the plot and was found to be equal to 7. 

The implementation of all preprocessing steps, along with the identification of the DE genes and cluster analysis, was performed in the Matlab^® ^7.9 (R2009b) (The MathWorks Inc. Natick, MA, USA) computing environment.

### Gene Ontology based analysis

In order to compare different groups of genes, highlighting different functionalities among all experimental setups, interesting genes formed study sets that were further subjected to Gene Ontology (GO) based analysis to test the nature of the observed resistance mechanism. Specifically, for each study set formed, statistical analysis of GO term overrepresentation was performed against the CGS, which was utilized as a reference set, as proposed by [55]. The chosen approach was the parent-child-union method [56], since it was found to outperform the standard method of overrepresentation analysis (ORA) in GO. The standard approach treats each GO term independently and hence does not take dependencies between parent and child terms into account, ignoring the structure of the GO hierarchy. It was shown that this behavior can result in certain types of false positive results, with potentially misleading biological interpretation [56]. In contrast, the parent-child method measures the overrepresentation of a term with respect to the presence of its parental terms in the set, and hence resolving the problem of the standard method which tends to falsely detect an overrepresentation of more specific terms below terms known to be overrepresented.

ORA was performed with the publicly available Ontologizer 2.0 tool [57] using GO terms definitions and associations between genes and GO downloaded from the Gene Ontology consortium [58] on the 26th of November 2010.

### TFBMs analysis

In order to identify the transcription factors driving the observed changes in the gene expression, we investigated the Transcription Factor Binding Motifs (TFBMs) in the Transcription Element Listening System Database (TELiS) [59]. The TRANSFAC transcription factor database was used for the identification of gene transcription factor binding sites [60].

### Chromosome mapping

Chromosome mapping appears to be a promising method for identifying patterns among genes. The main idea reported initially by [61] is to map genes in chromosomal regions and in this way, if correlations do exist, they appear through the location of genes on chromosomal regions, since consistent expression of whole functional entities is related with activation of given chromosomal regions. For chromosome mapping analyses, we used the Gene Ontology Tree Machine, WebGestalt web-tool [62].

### Pathway analysis

Pathway analysis was performed in order to find genes that participate in known pathways related to the investigated mechanisms, such as apoptosis or cell proliferation. This approach was used in order to gain further insight into the mechanisms underlying the common genes identified by our proposed model. The differentially expressed genes were mapped on different pathways using the Pathway Explorer software [63]. First of all, it was investigated the percentage of genes present in all known pathways using the databases available through the Pathway Explorer software. The KEGG Pathways database was used for our analysis [64-66].

### Hypothesis examination and computational work flow

#### Hypothesis statement

In order to answer the question of inherently disposed or acquired resistance and examine our initial working hypothesis, significant genes derived from the computational workflow were grouped in three sets, for each experiment *i*, with *i *= 1...5, *S_i_*, *U_i_*, and *D_i_*, where: *S_i _*includes genes which expression is considered to be unchanged, *U_i _*includes genes up-regulated, and *D_i _*includes genes down-regulated. Furthermore, in order to facilitate interpretation, gene functions were categorized in two major categories, *F_p _*and *F_a_*, where *F_p _*is related to apoptosis evasion and cell cycle progression, whereas *F_a _*is related to apoptosis induction and cell cycle arrest. In other words, *F_p _*is related to genes that are not desired targets of prednisolone regulation and the glucocorticoid receptor (GR) in the system under study, while *F_a _*is related to genes involved in apoptosis, which are the desired ones of GC regulation.

#### Modeling approach

**Figure 1:**
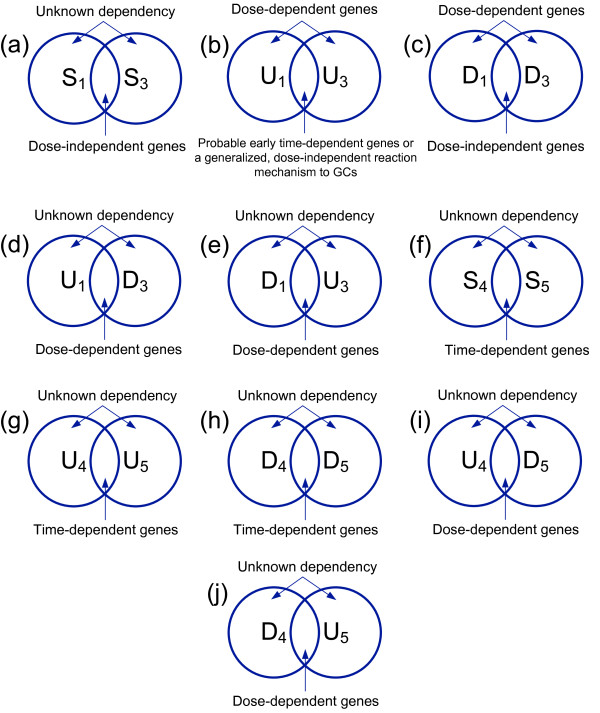
**Venn diagrams illustrating the intersections of the formulations posed for resistance**. Schematic representation of the set intersections (a) - (j) formed based on the definitions described in Methods, in order to answer the question of inherently disposed or acquired resistance. Sets *S_i_*, *U_i _*and *D_i _*include genes constantly expressed, up-regulated, and down-regulated in experiment *i*, respectively.

Concerning the 4 h treatment, namely the early treatment, the following functional categories were defined:

i) If genes simultaneously remain unaffected at the low and high prednisolone doses (intersection of sets *S*_1 _and *S*_3 _(Figure 1a), which points out dose-independent genes), and GO functions are related to *F_p_*, then those functions seem not to confer to the manifestation of resistive behavior by the cells, thus suggesting that these cells are originally sensitive and gradually develop resistant phenotypic characteristics. On the contrary, if gene functions are related to *F_a _*then cells tend to remain unresponsive to GC treatment, thus implying an intrinsic mechanism of resistance.

ii) If genes are simultaneously up-regulated at both concentrations (intersection of sets *U*_1 _and *U*_3 _(Figure 1b), which points out probable early time-dependent genes or a generalized reaction mechanism to GCs which is dose-independent), and GO functions are related to *F_p_*, then cells have an intrinsic mechanism of resistance. On the contrary, if gene functions are related to *F_a _*then cells are originally sensitive and gradually develop, or homeostatically adjust to resistant phenotypic characteristics.

iii) If genes are simultaneously down-regulated at the low and high prednisolone doses (intersection of sets *D*_1 _and *D*_3 _(Figure 1c), which points out dose-independent genes, that manifest the same expression profile under these two extreme GC concentrations, viz, down-regulated in the cell system in its natural condition), and GO functions are related to *F_p_*, then cells are originally sensitive and gradually develop resistant phenotypic characteristics. On the contrary, if gene functions are related to *F_a _*this predicates upon an intrinsic mechanism of resistance by the cells.

iv) If genes are simultaneously up-regulated at the low prednisolone concentration and down-regulated at the high prednisolone dose (intersection of sets *U*_1 _and *D*_3 _(Figure 1d), which points out the dose-dependent genes) and GO functions are related to *F_p_*, then cells tend to present a dose dependent sensitization to GC exposure, thus postponing resistance manifestation at a later point, something which implies acquired resistance mechanisms. On the contrary, if gene functions are related to *F_a _*then cells have an intrinsic mechanism of resistance.

v) Finally, if genes are simultaneously down-regulated at the low prednisolone concentration and up-regulated at the high prednisolone dose (intersection of sets *D*_1 _and *U*_3 _(Figure 1e), which points out the dose-dependent genes) and GO functions are related to *F_p_*, then cells show a dose-dependent intrinsic mechanism of resistance. On the contrary, if gene functions are related to *F_a _*then cells present dose-dependent sensitization, postponing resistance manifestation at a later point something which implies acquired resistance mechanisms.

Further on, extrapolating the aforementioned approach in the analysis of the 72 h experiments, namely the late treatment, the following functional categories were defined:

i) If genes remain unchanged (intersection of sets *S*_4 _and *S*_5 _(Figure 1f) or are up-regulated (intersection of sets *U*_4 _and *U*_5 _(Figure 1g) at both treatments at 72 h, and GO functions are related to *F_p_*, then cells manifest a time-dependent acquired mechanism of resistance. If the opposite is true, then the nature of the resistance mechanism cannot be defined.

ii) If genes are down-regulated (intersection of sets *D*_4 _and *D*_5 _(Figure 1h) and GO functions are related to *F_a_*, then the mechanism of resistance is probably inherent. Again, if the opposite is true, then the nature of the resistance mechanism cannot be defined.

iii) If genes are up-regulated at the 22 uM vs. 700 uM experiment and down-regulated at the control vs. 700 uM experiment (intersection of sets *U*_4 _and *D*_5 _(Figure 1i) or are down-regulated at the 22 uM vs. 700 uM experiment and up-regulated at the control vs. 700 uM experiment (intersection of sets *D*_4 _and *U*_5 _(Figure 1j), and GO functions are related to *F_p_*, then cells manifest an acquired resistance mechanism which is partially remitting to a dose-dependent re-sensitization by large GC doses. On the other hand, if gene functions are related to *F_a_*, then the mechanism of resistance cannot be defined.

**Figure 2:**
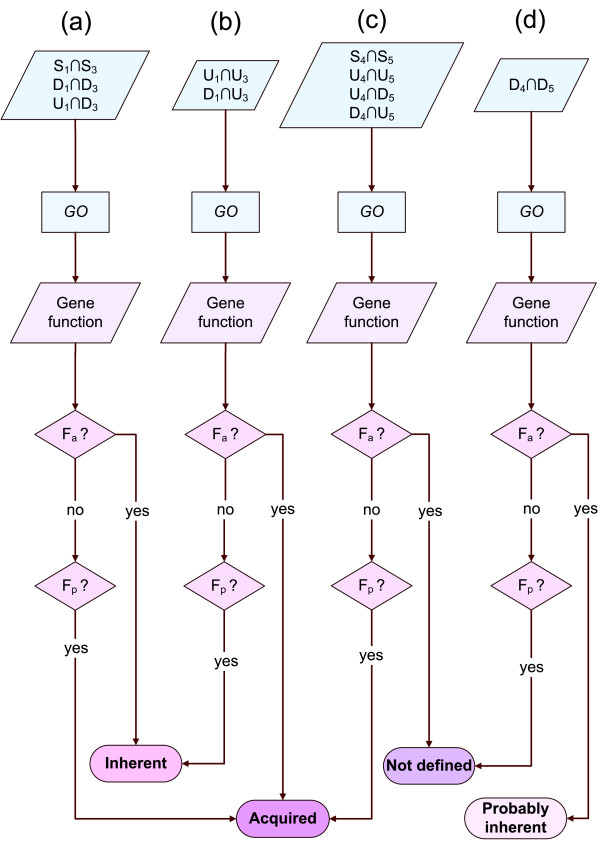
**Flowcharts of the hypotheses posed for resistance**. Simplified flowcharts (a) - (d) of the hypotheses posed for inherent or acquired resistance in ALL cells. Subsets *S*_1 _∩ *S*_3_, *D*_1 _∩ *D*_3 _and *U*_1 _∩ *D*_3 _follow flowchart (a), subsets *U*_1 _∩ *U*_3 _and *D*_1 _∩ *U*_3 _follow flowchart (b), subsets *S*_4 _∩ *S*_5_, *U*_4 _∩ *U*_5_, *U*_4 _∩ *D*_5 _and *D*_4 _∩ *U*_5 _follow flowchart (c), and subset *D*_4 _∩ *D*_5 _follows flowchart (d). Sets *S_i_*, *U_i_*, and *D_i _*include genes constantly expressed, up-regulated, and down-regulated in case *i*, respectively, where subscript *i *is varying from 1 to 5, and refers to each experimental setup. Each subset is tested for its GO enrichments, while gene functions are categorized as *F_a_*, *F_p_*, where *F_a _*is related to apoptosis induction and cell cycle arrest and *F_p _*is related to apoptosis evasion and cell cycle proliferation.

All aforementioned combinations are summarized in the simplified flowcharts of Figure 2.

## Results

Gene expression profiling concerning two distinct time points (4 h and 72 h) followed mRNA isolation from the CCRF-CEM cell line, cultured in three different prednisolone concentrations (10 nM, 22 uM, and 700 uM). The mRNA collected was used for hybridization on two cDNA microarray platforms (1.2 k and 4.8 k). Specifically, the experimental design consisted of the five following pairs: control vs. 10 nM prednisolone at 4 h (designated as '1'), 10 nM vs. 700 uM prednisolone at 4 h (designated as '2'), control vs. 700 uM prednisolone at 4 h (designated as '3'), 22 uM vs. 700 uM prednisolone at 72 h (designated as '4'), and control vs. 700 uM prednisolone at 72 h (designated as '5').

A common for both platforms data preprocessing stage was applied to the raw expression datasets, including proper background correction for each replicate slide, within-slide normalization and filtering. Concerning data integration, a two-step procedure was applied, firstly by matching reporters on the two, microarray platforms and then applying a cross-platform, normalization approach. In this way the preprocessed expression values of the common gene-level identifiers, existing in both platforms, were selected to form a unified dataset on which standard statistical procedures could be applied. Each subsequent analysis step was performed on the integrated gene expression values of this unified dataset (Additional file 3), which includes a total number of 490 genes, common in all experimental setups (see Methods for details).

### Gene Ontology analysis based on statistically significant genes

In order to identify differentially expressed (DE) genes for each experiment, we utilized the intensity-dependent calculation of the standard Z-score [32] of each common feature within each slide, at a confidence level of 95%. Then, for each experiment *i*, with *i *= 1...5, all genes were further classified into the three sets: *S_i_*, *U_i_*, and *D_i_*, where *S_i _*includes genes which expression is considered to be unchanged, *U_i _*includes genes up-regulated, and *D_i _*includes genes down-regulated (Additional file 4).

#### Set intersections

In order to address the question of the prevalence of either inherently disposed or acquired mechanisms of resistance, the following intersections were defined, in line with the formulations described in the *Methods *section (Figure 1): *S*_1 _∩ *S*_3_, *U*_1 _∩ *U*_3_, *D*_1 _∩ *D*_3_, *U*_1 _∩ *D*_3_, *D*_1 _∩ *U*_3_, *S*_4 _∩ *S*_5_, *U*_4 _∩ U_5_, *D*_4 _∩ *D*_5_, U_4 _∩ *U*_5_, and *D*_4 _∩ *U*_5 _(Additional file 4). Then, these intersections were subjected to statistical analysis of *Gene Ontology *(*GO*) term overrepresentation, in order to test the nature of the observed resistance mechanism (Additional file 5). The generated from the *GO*-based analysis gene functions were also further categorized in two major categories: *F_p _*and *F_a_*, where *F_p _*is related to apoptosis evasion and cell cycle progression, whereas *F_a _*is related to apoptosis induction and cell cycle arrest (Figure 2).

The results of this examination demonstrated specifically that:

i) Genes belonging to intersection *S*_1 _∩ *S*_3 _(Figure 1a) do not incorporate any functions related to apoptosis induction (*F_a_*) or cell proliferation (*F_p_*), thus the nature of the resistance mechanism cannot be defined.

ii) No common genes were observed for sets *U*_1 _and *U*_3 _(Figure 1b), implying that no genes from the present dataset are dose-independent upon prednisolone treatment.

iii) Intersection *D*_1 _∩ *D*_3 _(Figure 1c) showed only one common gene, the *DAPK1*. However, DAPK1 is known to be a positive mediator of gamma interferon induced cell death [67], thus down-regulation of *DAPK1 *implies a possible linkage with inherent resistance mechanisms. Moreover, it appears that *DAPK1*'s down-regulation is dose-independent and the glucocorticoid receptor (GR), when stimulated in the system under study, deactivates it.

iv) Intersections *U*_1 _∩ *D*_3 _(Figure 1d) and *D*_1 _∩ *U*_3 _(Figure 1e) do not yield common genes. Since there are no genes that are regulated dose-dependently and in opposing manners, it may be concluded that, at least as far as the present dataset is concerned, prednisolone regulates different sets of genes in a dose-dependent manner.

Regarding the 72h time point:

i) Genes belonging to intersection *S*_4 _∩ *S*_5 _(Figure 1f) comprised functions related to induction of apoptosis and cell cycle arrest (*F_a_*), thus they do not confer any knowledge about the nature of the resistance mechanism.

ii) Intersections *U*_4 _∩ *U*_5 _(Figure 1g) and *D*_4 _∩ *D*_5 _(Figure 1h) did not yield common genes, whereas intersection *U*_4 _∩ *D*_5 _(Figure 1i) captured one gene, namely the gene *KIT*. This gene is reported to play a role in a variety of human tumors, including acute myelogenous leukemia, in its mutated forms [68]. From the present study it appears that it is also active in the regulation of GC action.

iii) Finally, one gene is derived from the intersection of *D*_4 _∩ *U*_5 _(Figure 1j), namely *MADD *(also known as *IG20*), which represents a very interesting gene since it has an established role in the mediation of the death signal from TNF-alpha downwards to the apoptosis activators [69]. In general, it is also known to be expressed at higher levels in neoplastic cells than in normal cells [70], which is also the case for our system. However, it is unclear if *MADD *over-expression in tumor cells incurs increased apoptosis or not. The case of this specific gene becomes more interesting as it is known that at least six different splice variants with equally different functions are expressed, each one performing different functions, both apoptotic and anti-apoptotic [70].

#### Individual sets

From the aforementioned it seems that dose-dependent prednisolone administration induces varying expression of different sets of genes. In this sense, a plausible presumption is that genes differentially expressed by prednisolone should to a large extent be implicated in the resistance mechanisms, responding to prednisolone treatment. Thus, apart from the aforementioned intersections, the sets *U*_1_, *U*_3_, *D*_1 _and *D*_4 _for the 4 h time point, and the sets *U*_4_, *U*_5_, *D*_4 _and *D*_5 _for the 72 h time point were also examined (Additional file 5). More specifically:

i) Gene set *U*_1_, concerning response to low dose GC administration was related to apoptotic and cell cycle regulation mechanisms. It is worth noting that this set (upregulated by 10 nM prednisolone) includes gene *BCL2L2*, which has been reported to be an anti-apoptotic gene [71].

ii) Gene set *U*_3_, concerning genes overexpressing as a result of high GC presence, did not highlighted functions relevant to apoptosis induction or positive regulation of cell death (*F_a_*). In this sense, it provides evidence for an inherent mechanism of resistance, since it seems that the high dose does not comply with the expected apoptotic actions. Interestingly, the same concentration (700 uM) seemed to stir metabolic as well developmental mechanisms.

iii) Both sets *D*_1 _and *D*_3 _did not incorporate functions related to apoptosis induction and positive cell death regulation (*F_a_*). In other words, the genes down-regulated both in low or high doses of prednisolone administration are not related to apoptosis induction. Nevertheless in set *D*_1_, gene *DAP*, which encodes for a protein that is positive regulator of programmed cell death [72], is related in addition to the process of autophagy. Moreover, this gene is a member of the mTOR pathway, which is at the same time, a negative regulator of autophagy [73]. Again, it appears that 10 nM prednisolone stimulates cell proliferation in addition to an anti-apoptotic effect. It cannot be precluded that prednisolone could potentially enhance alternative nutrition mechanisms as an alternative way to attain survival. It appears that GC presence is impacting the way the cell will metabolize.

Regarding the 72h time point:

i) It appears that prednisolone exerts a diversified intricate mechanism as it up-regulates (*U*_4_) genes, such as *BCL2*, which is an anti-apoptotic gene. Moreover, functions were revealed that had to do with positive regulation of locomotion, indicating the activation of a possible mechanism of evasion from a hostile environment.

ii) Also, prednisolone up-regulated genes related to apoptosis evasion (*F_p_*) at the 700 uM dose as compared to untreated cells (*U*_5_), among these, gene *BIRC5*. The *BIRC *family of genes belongs to the larger *IAP *family of genes (inhibitors of apoptosis genes). *BIRC5 *especially is considered to be an apoptosis evasion factor with significant presence in a variety of tumors [74]. The fact that prednisolone up-regulates such a gene, supports the case of an acquired mechanism of action.

iii) In addition, prednisolone down-regulated genes (*D*_4 _and *D*_5_) are related to stimulation of cell cycle progression (*F_p_*), such as *BCL2L2 *and *KIT*, respectively. Also, genes related to positive induction of cell death, such as *IKBKE *and *PPP3CC *[40] are down-regulated. However, it is worth mentioning that there is also a shift of molecular function towards NF-κB-related effects. Especially, gene *IKBKE*, which is a non-canonical Iκ-B kinase, a known controller of NF-κB [75], appears to be down regulated by prednisolone at 72 h. This is the same gene that is involved in induction of cell death, i.e. IKBKE, which is a non-canonical Iκ-B kinase, a known controller of NF-κB [75].

A very interesting case is presented in these clusters with the MCL1 gene. As mentioned before, this gene is instrumentally linked to cell survival and the fact that the low dose activates it, is a strong indication for inherent resistance in the present system. This indication becomes stronger by the finding that this gene is similarly expressed in untreated cells and cells treated with various concentrations of prednisolone. As a matter of fact it seems that even variations in concentrations such as the 22uM and 700uM do not affect its expression, at least at late time points, or its expression has been stabilized. In cluster 7, gene functions appeared to be related to cell cycle regulation. This group of clusters seem to outline the effects of the high prednisolone dose, as this dose activates genes related to cell cycle progression. For example, in these clusters appear genes such as BMP5, FGF7 and MCL1. Those genes, are promoters of anti-apoptosis and proliferation. Over-expression of such genes, at later time intervals, points out the existence of an inherent mechanism of reaction to the glucocorticoid. Yet, the fact that their expression at 4h remains stable, implies that for this gene set the decision for gene activation or not is taken later on during GC treatment. The fact however, is that cells react with anti-apoptotic signals already at 4h and our hypothesis is that the 72h activation of anti-apoptotic genes has to do with a re-enforcement of the resistant phenotype that the cells already possess. 

Taken together the results of all the studied subsets, summarized in Table 1, showed that the cell system under study exerts an intricate response pattern of inherent and acquired mechanisms of GC resistance, which seems however to favor inherent resistance mechanisms.

**Table 1:** Summary of the GO functions revealed for each gene set

Gene Group^a^		**No**.	Gene^b^	Gene Function^c^				Resistance^d^	*p*-value^e^
				*F_p_*	*F_a_*	*F_m_*	*F_i_*		
4 h	*S_1 _*∩ *S_3_*	423		no	no	no	yes	n/d	< 0.05
	*U_1 _*∩ *U_3_*	0		n/a	n/a	n/a	n/a	n/a	n/a
	*D_1 _*∩ *D_3_*	1	*DAPK1*	no	yes	no	no	inherent	< 0.05
	*U_1 _*∩ *D_3_*	0		n/a	n/a	n/a	n/a	n/a	n/a
	*D_1 _*∩ *U_3_*	0		n/a	n/a	n/a	n/a	n/a	n/a
	*U_1_*	12	*BCL2L2*	yes	no	no	no	inherent	< 0.05
	*U_3_*	11		yes	no	yes	no	inherent	< 0.05
	*D_1_*	23	*DAP*	no	yes	yes	no	inherent	< 0.05
			*CASP1*	no	yes	no	no	inherent	< 0.05
			*CDC25*	yes	no	no	no	acquired	< 0.05
	*^D^3*	22	*IL24*	no	yes	no	no	inherent	< 0.05

72 h	*S_4 _*∩ *S_5_*	433		no	yes	yes	no	n/d	< 0.05
	*U_4 _*∩ *U_5_*	0		n/a	n/a	n/a	n/a	n/a	n/a
	*D_4 _*∩ *D_5_*	0		n/a	n/a	n/a	n/a	n/a	n/a
	*U_4 _*∩ *D_5_*	1	*KIT*	yes	no	no	no	acquired	< 0.05
	*D_4 _*∩ *U_5_*	1	*MADD*	n/d	n/d	n/d	n/d	n/d	< 0.05
	*U_4_*	9	*BCL2*	yes	no	no	no	acquired	< 0.05
	*U_5_*	12	*BIRC5*	yes	no	no	no	acquired	< 0.05
	*D_4_*	19	*BCL2L2*	yes	no	no	no	acquired	< 0.05
	*D_5_*	18	*IKBKE*	no	yes	no	yes	~ inherent	< 0.05
			*PPP3CC*	no	yes	no	no	~ inherent	< 0.05

clusters	*^C^1*	4		n/a	n/a	n/a	n/a	n/a	n/a
	*^C^2*	11		n/d	n/d	n/d	n/d	n/a	n/a
	*^C^3*	14		no	yes	no	no	inherent	< 0.05
	*^C^4*	25		n/a	n/a	n/a	n/a	n/a	n/a
	*^C^5*	17		yes	yes	no	no	n/d	< 0.05
	*^C^6*	10		no	yes	no	no	inherent	< 0.05
	*^C^7*	19		yes	no	yes	no	acquired	< 0.05

^a ^*S_i_*, *U_i_*, and *D_i _*correspond to groups which include genes constantly expressed, up-regulated, and down-regulated in experiment *i*, respectively, and *C_i _*to a cluster of genes showing similar expression profile. Subscript *i *is varying from 1 to 5 and refers to each experimental setup, ^b ^Selected genes from each gene set, ^c ^*F_p _*= is related to apoptosis evasion and cell cycle progression, *F_a _*= is related to apoptosis induction and cell cycle arrest, *F_m _*= is related to metabolic processes, *F_i _*= is related to the regulation of IκB/NFκB, n/a = not available, and n/d = not defined, ^d ^~ inherent = probably inherent, ^e ^Each *p*-value corresponds either to the one-sided Fisher exact test of the GO-based analysis of the respective gene set, or, in case of a single gene, to the intensity-dependent Z-score.

We have seen that at early time points, there are no genes, which are regulated dose-dependently or in opposing manners. Therefore, we concluded that prednisolone regulates different sets of genes in a dose-dependent manner, at least as far as the present data set is concerned. This helped us to conclude that genes differentially expressed by prednisolone should play a role in the resistance mechanism to prednisolone.

### Gene Ontology analysis based on clustering

**Figure 3:**
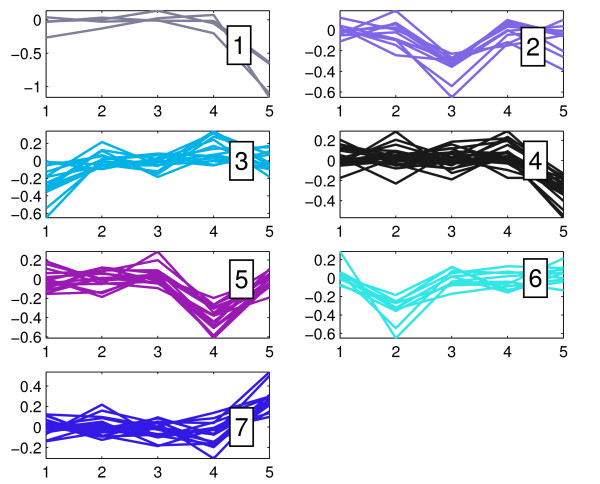
**Expression profile clustering**. K-means clustering of the gene expression profiles illustrating the seven clusters as described in Methods. Each subplot corresponds to one cluster of genes showing similar expression profile. The horizontal axes correspond to the five prednisolone experiments of interest, where '1' = control vs. 10 nM prednisolone at 4 h, '2' = 10 nM vs. 700 uM prednisolone at 4 h, '3' = control vs. 700 uM prednisolone at 4 h, '4' = 22 uM vs. 700 uM prednisolone at 72 h, and '5' = control vs. 700 uM prednisolone at 72 h. The vertical axes depict the corresponding logarithmic ratios, as derived from microarray data analysis.

K-means clustering is an effective way of classifying gene expression profiles, based on their similarities as well as on probable common regulatory mechanisms. In this way, it can be considered as an effective way to suggest candidate targets of putative, common regulatory mechanisms. Thus, as described in Methods, the top 100 genes with the highest SD (SD100) from the CGS was subjected to k-means clustering using squared Euclidean as a distance measure, in order to identify groups of genes presenting similar expression profiles during prednisolone treatment early and late response and possibly co-regulated. In Figure 3, the k-means clustering is presented, illustrating the seven clusters derived (Additional file 6). GO-based enrichment analysis, that followed, linked co-expressed genes in each cluster to several functional categories (Additional file 5). In particular, we noted that:

i) Cluster 1 (*C*_1_) contains time-dependent genes, which expression decreases at 700 uM prednisolone and the 72 h time point (resembling the behaviour of the *D*_5 _set), while remaining unchanged at all other conditions (including genes from the *S*_1 _∩ *S*_2 _set). However, genes under this cluster did not yield any significant *GO *terms.

ii) Cluster 2 (*C*_2_) mainly comprises genes showing early down-regulation at 700 uM prednisolone (including genes from the *D*_3 _set), while remaining unchanged at all other conditions (probably outlining genes of the *S*_4 _∩ *S*_5 _set). *GO*-based analysis revealed genes involved in developmental processes, implying a possible existence of stem cell related functions in the cell system under study.

iii) Cluster 3 (*C*_3_) depicts genes showing early down-regulation at 10 nM prednisolone (including genes from the *D*_1 _set), while remaining unaffected by the 700 uM prednisolone treatment. This cluster comprised genes related to positive regulation of cell death. The fact that these genes are suppressed under low prednisolone concentration at the early time point is in agreement with the anti-apoptotic and survival effect reported previously by [76], which implies an intrinsic mechanism of resistance.

iv) Cluster 4 (*C*_4_), similarly to (*C*_1_), presents genes which expression at 700 uM prednisolone decreases at the 72 h time point (resembling the behaviour of the *D*_5 _subset), while remaining unchanged at all other conditions (including genes from the *S*_1 _∩ *S*_3 _subset). However, genes under this cluster did not present any interesting *GO *terms.

v) Cluster 5 (*C*_5_) contains genes remaining unchanged at all conditions (including genes from the *S*_1 _∩ *S*_3 _intersection) except for experimental setup '4', where they are down-regulated (resembling the behaviour of the *D*_4 _set). *GO*-based analysis of cluster 5 revealed genes that are involved in the opposing processes of cell cycle progression, as well as cell cycle arrest, and cell death functions.

vi) Cluster 6 (*C*_6_) depicts genes that are also unchanged at all conditions (including genes from the *S*_4 _∩ *S*_5 _intersection) except for experimental setup '2', where they seem to be down-regulated (resembling the behaviour of the *D*_2 _set). Functions represented by genes in cluster 6 include cell death functions so their down regulation at the specific setup supports the case of inherent resistance mechanisms.

vii) Finally, cluster 7 (*C*_7_) groups together time-dependent genes that remain unaffected at all experimental setups (including genes from the *S*_1 _∩ *S*_3 _intersection), but at 700 uM after 72 h of treatment (resembling the behaviour of the *U*_5 _set), where they are positively regulated. It appears that those genes participate in cell cycle regulation. This cluster seems to outline the effects of the high prednisolone dose, as this dose activates genes related to cell cycle progression. Special attention was drawn to the MCL1 gene. This gene is a member of the *BCL2 *family and it produces an anti-apoptotic protein responsible for cell survival.

The cluster analysis results, summarized in Table 1, also confirm that, although the gene expression profiles of the cell system under study present a mixed response of inherent and acquired mechanisms of GC resistance, however there is a tendency towards inherent resistance mechanisms.

### TFBM analysis

A next step on our analysis was the identification of common transcription factors that would regulate genes in a similar way. Cluster 1 (*C*_1_) manifested a common transcription factor namely OCT1 (*p *= 5 · 10^-5^). Cluster 2 (*C*_2_) similarly manifested a common transcription factor, MZF1 (*p *= 8 · 10^-4^). Cluster 5 (*C*_5_) were predicted to be commonly regulated by the CCAAT (NFYA) transcription factor (*p *= 5 · 10^-7^). Cluster 6 (*C*_6_) presented an interesting case, as the common most prevalent transcription factor was JUN. Finally, Cluster 7 (*C*_7_) also manifested different transcription factors. In particular, the most prevalent transcription factor was OCT1 (*p *= 1 · 10^-10^).

### Chromosome mapping

**Figure 4:**
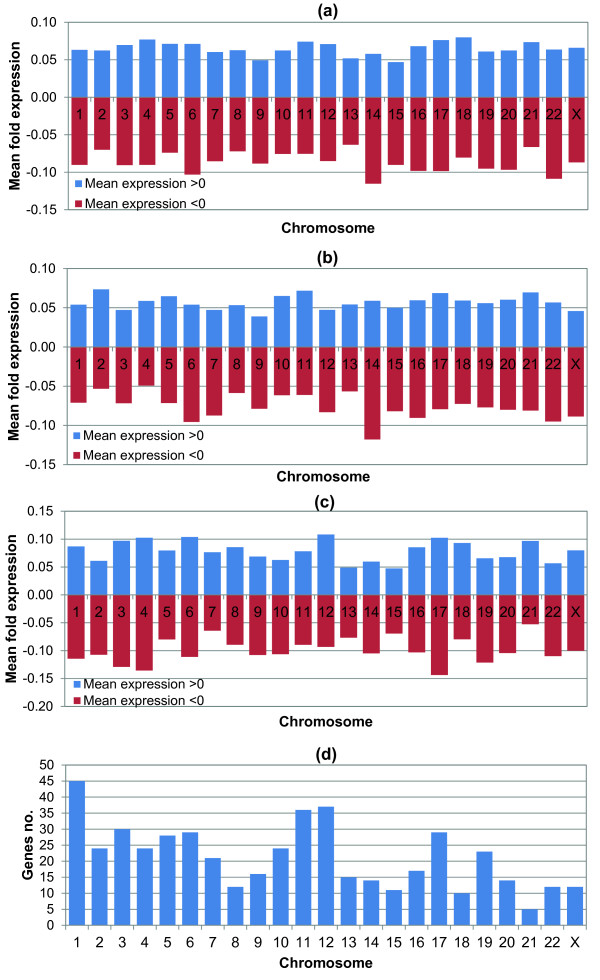
**Chromosome-related gene expression**. The mean values of all genes at all time points (a) is presented separated in positive and negative fold expression. Chromosomes 14 and 22 showed to have the most deactivated genes as compared to untreated cells. Similarly, mean gene expression has been separated to the 4 h (b) and 72 h treatments (c). Further on we have searched whether mean gene expression is associated with the represented genes on each chromosome as the number of genes is presented on (d).

In order to find further patterns of expression in the system under study, we have performed a chromosome mapping analysis, where the mean expression of genes with respect to chromosomes has been studied. A general remark from this analysis is that expression seemed to be equivalently distributed; at least as far as positive regulation is concerned, while chromosomes 14 and 22 appeared to have genes with most deactivation as compared to untreated genes (Figure 4a).

**Figure 5:**
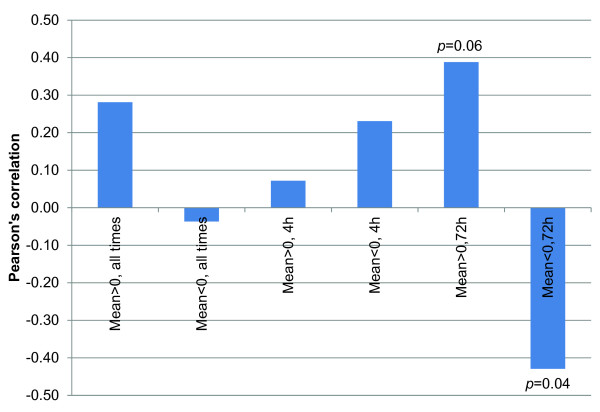
**Pearson's correlation values comparing gene numbers to mean gene expression**. Graphical illustration of Pearson's correlation values between gene numbers represented on each chromosome and the mean expression values for experimental setups. This analysis showed no significant results except for the case where negative gene expression is correlated to gene number. That means that the more genes are represented on a chromosome the less these are expressed.

Further on, we have separated gene expression based on the time points i.e. we have calculated the mean expressions for the 4 h and 72 h treatments (Figure 4b and 4c, respectively). We have tried to identify whether the mean expression values, either positive or negative, correlate to the number of genes represented on each chromosome. In Figure 4d, the number of genes per chromosome is presented. Table 2 presents the calculated correlation values of mean expression values for experimental time points, while Figure 5 graphically illustrates the Pearson's correlation values comparing gene numbers to mean gene expression. Interestingly, gene number is negatively correlated to negative mean expression, which means that the more genes are represented on chromosomes the more these genes are down-regulated as compared to untreated cells or, in order to be more concise, the more these genes are inactivated as compared to control.

**Table 2:** Pearson's correlation analysis of the mean expression values of genes with respect to their chromosome allocation

Pearson's correlation value^a^								
			All		4 h		72 h	
		**Genes no**.	m_M _> 0	m_M _< 0	m_M _> 0	m_M _< 0	m_M _> 0	m_M _< 0
	**Genes no**.	1.0000	0.2813	-0.0368	0.0719	0.2309	0.3883	-0.4294
**All**	**m_M _> 0**	0.2813	1.0000	0.0383	0.4537	0.1325	0.7978	-0.2066
	**m_M _< 0**	-0.0368	0.0383	1.0000	0.2503	0.7349	-0.0249	0.5293
**4 h**	**m_M _> 0**	0.0719	0.4537	0.2503	1.0000	0.2789	0.0053	-0.0059
	**m_M _< 0**	0.2309	0.1325	0.7349	0.2789	1.0000	0.0385	-0.0599
**72 h**	**m_M _> 0**	0.3883	0.7978	-0.0249	0.0053	0.0385	1.0000	-0.2422
	**m_M _< 0**	-0.4294	-0.2066	0.5293	-0.0059	-0.0599	-0.2422	1.0000
***p*-value^a^**								
			**All**		**4 h**		**72 h**	
		**Genes no**.	**m_M _> 0**	**m_M _< 0**	**m_M _> 0**	**m_M _< 0**	**m_M _> 0**	**m_M _< 0**
	**Genes no**.	0.0000	0.1936	0.8677	0.7444	0.2891	0.0671	0.0409
**All**	**m_M _> 0**	0.1936	0.0000	0.8624	0.0297	0.5467	0.0000	0.3441
	**m_M _< 0**	0.8677	0.8624	0.0000	0.2493	0.0001	0.9101	0.0094
**4 h**	**m_M _> 0**	0.7444	0.0297	0.2493	0.0000	0.1975	0.9808	0.9786
	**m_M _< 0**	0.2891	0.5467	0.0001	0.1975	0.0000	0.8616	0.7859
**72 h**	**m_M _> 0**	0.0671	0.0000	0.9101	0.9808	0.8616	0.0000	0.2655
	**m_M _< 0**	0.0409	0.3441	0.0094	0.9786	0.7859	0.2655	0.0000

^a ^*m_M _*= mean gene expression.

### Pathway analysis

**Figure 6:**
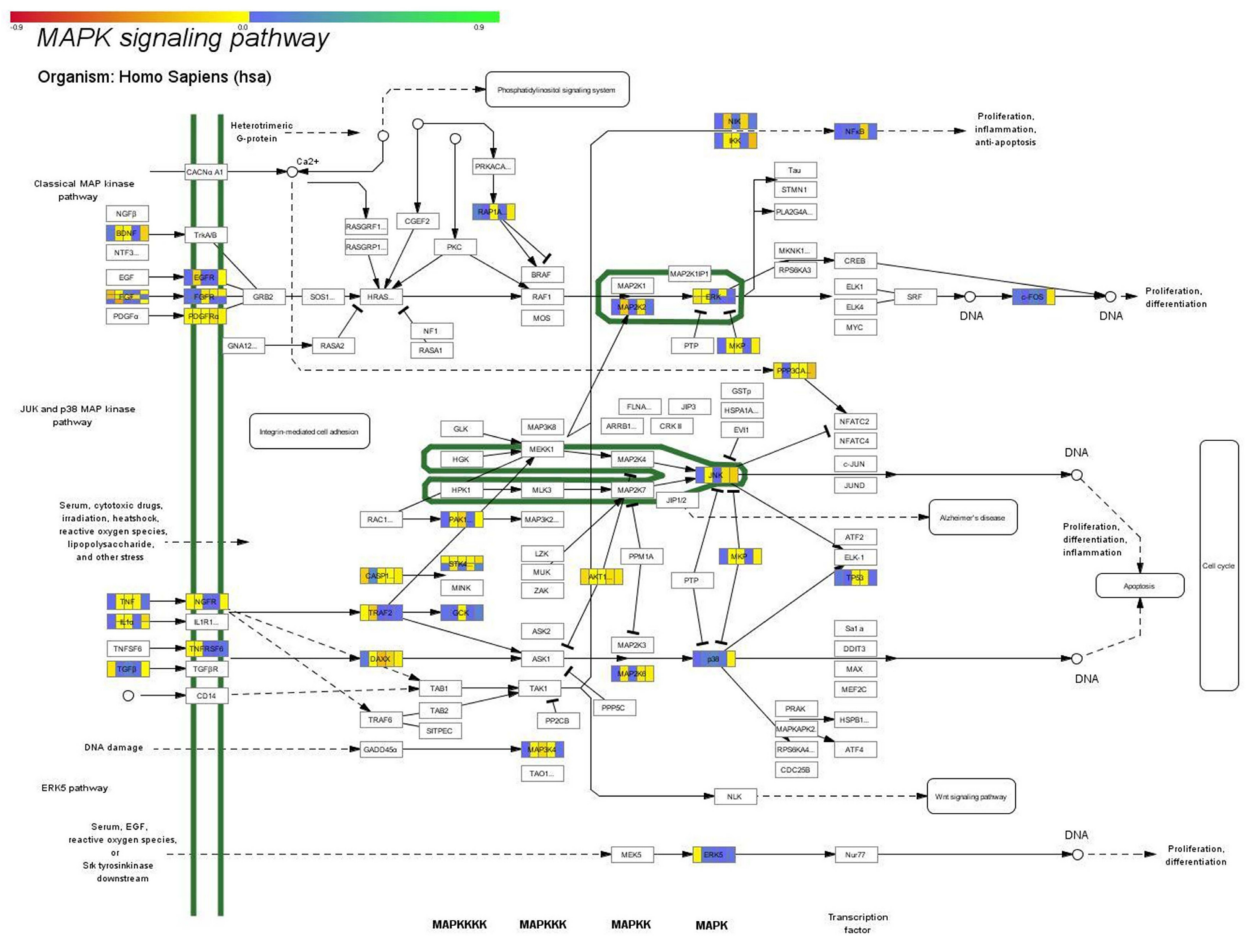
**Mapped genes of the common dataset on the KEGG MAPK pathway**.

**Figure 7:**
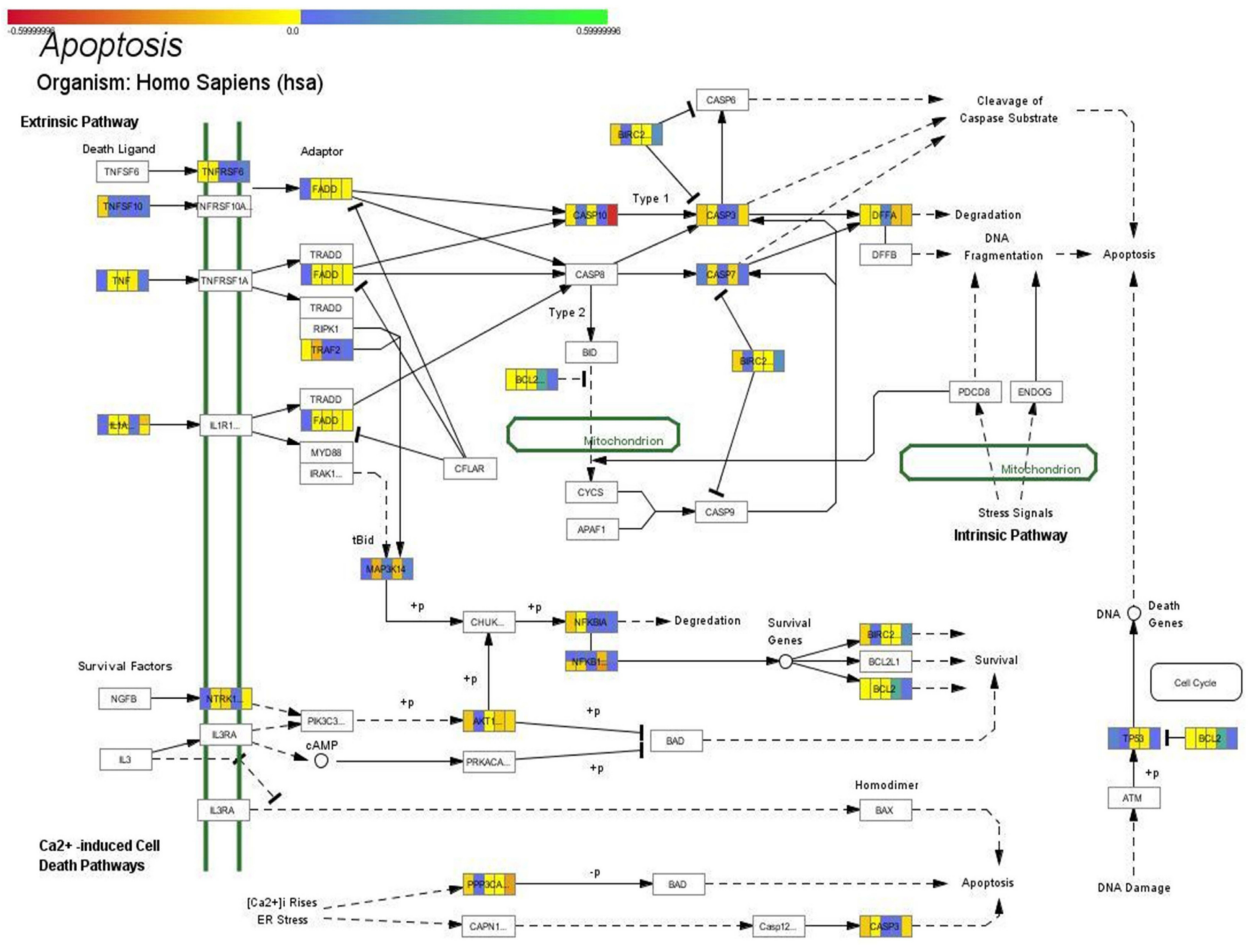
**Mapped genes of the common dataset on the KEGG apoptosis pathway**.

In order to gain further insight into the possible mechanisms that probe for the preponderance of inherence or acquired resistance mechanisms, we have attempted to perform a pathway analysis with the defined genes from our dataset. We have mapped the common dataset on known pathways and in particular on the KEGG Pathways database. In particular, we have outlined two pathways: the MAPK pathway, as it incorporated the larger number of involved genes to be mapped on it (Figure 6), and the apoptosis pathway, since we were interested in examining the possible role of genes related to apoptotic mechanisms (Figure 7).

It is known that the MAPK signaling pathway is involved in cell cycle progression and apoptosis. We have outlined several cross-talking molecules in this pathway that were revealed also by our dataset, such as the NF-κB, JNK, p38 and ERK5. NF-κB remains stable across the experimental setups indicating a steady role in apoptosis. Yet, as it is regulated by the GR then its levels should be lowering as concentration increases. This hints towards a more specific, more pronounced involvement of the NF-κB factor in the observed resistance. At the same time, the JNK kinase, which is a mediator of apoptosis, appears to remain unaffected by the prednisolone concentrations indicating that the MAPK pathway involved in apoptosis does not function in the way it should have, hence supporting an inherent mechanism of resistance. Finally, an interesting molecule is the ERK5, which appears to be down-regulated at the low doses and unaffected thereafter. This molecule is involved in cell proliferation and its down-regulation is one of the very few implications that prednisolone behaves as expected, hence indicating an acquired mechanism of resistance.

Moving to the apoptosis pathway, several interesting genes that play a role in the observed apoptosis were identified. In particular, based on the signaling on Figure 7, we can discriminate between the TNF/FADD/CASP/BIRC system (top of the figure), where it appears that molecules responsible for degradation are down-regulated under prednisolone treatment. In particular, *BIRC5 *was found to be activated in the 72 h treatment, while remained unchanged in the previous time point. In addition, *CASP1 *was found to be down-regulated at 10 nM treatment and 4 h, indicating an inherent mechanism. This part of the pathway seems to function in such a way as cells already possess glucocorticoid resistance. The second part of the pathway consisting from NGBF/NTRK1/AKT1/NFκB/BIRC/BCL2 (family) shows the following. AKT1 appeared to remain unchanged at all concentrations and time points. NF-κB1 is down-regulated at the late time points, indicating a late response to the glucocorticoid, while it is unchanged at the early time points. The BCL family is a super-family of proteins that when expressed promotes survival. In the present dataset, the *BCL2 *gene was found to remain unchanged, while a member of the super-family, *BCL2L2*, was up-regulated. *BCL2L2 *is one of the genes that participate in survival in the pathway, confirming the fact that we have an early anti-apoptosis response. The role of late NFKB1 inactivation and early BCL2L2 activation probably favors an acquired mechanism of resistance. Finally, an apoptotic signaling avenue is via the regulation of Ca^+2 ^where the caspases participating in this pathway are inactivated in the present system. The PPP3CC protein that regulates phosphorylation of down-stream targets has a relation to apoptosis over the activation of CASP proteins. Both are inactivated in the present system with PPP3CC being inactivated at 72 h and CASP1 at 4 h. This presents a mixed mechanism of apoptosis evasion.

## Discussion

In the present work, we have set up and propose a rational computational analysis framework, in order to aid the elucidation of the molecular mechanisms of glucocorticoid resistance and specifically whether these are to a larger extent inherent or acquired. To the best of our knowledge, there are no reports trying to analyze systematically the underlying, inherent or acquired molecular mechanisms of GC resistance. The issue whether leukemic cells possess the inherent genetically imprinted resistance mechanisms or it is rather a post effect of evolutionary adoption of originally sensitive cells upon GC treatment is still a controversial one, with potentially significant interest for design of novel therapeutic approaches in cancer treatment. A similar work [77] reported recently that in *ex vivo *samples, leukemic cells exhibit an at least in part, intrinsic mechanism of reaction. In another work [78], it has also been mentioned that glucocorticoids can induce intrinsic mechanisms of GC-resistance such as the BCL2 relais.

The initial working hypothesis was that the *in vitro *system of the present study presents an inherent mechanism of reaction to glucocorticoids, resulting in resistance to apoptosis. Our results showed that cells may attain a mixed mechanism of response to glucocorticoids, however, there is clear evidence predicating towards an intrinsic mechanism of resistance.

Specifically, several interesting genes, whose expression is regulated by the GR, were identified. One of these genes was *MCL1*. This gene belongs to the *BCL *super-family, which is responsible for cell survival. It has been reported that down-regulation of *MCL1 *sensitizes T-cell leukemia cells to treatment with glucocorticoids [79]. In our system this gene appeared to be stable across all concentrations while, interestingly, it was up-regulated by the low prednisolone dose, confirming the anti-apoptotic effect observed previously by the low dose. At the same time another member of the *BCL *family, *BCL2L2*, also responsible for cell survival was up-regulated by the low dose of prednisolone. There are no reports connecting *BCL2L2 *with leukemia, yet some reports refer to its pro-survival role in leukemogenesis [80,81]. An interesting gene also revealed by the present analysis was *DAPK1*, a tumor suppressor gene, simultaneously down-reglulated at both prednisolone concentrations. Methylations of this gene have been linked to hereditary character of chronic leukemias [82,83] as well as its expression has been linked to childhood leukemia [84,85]. Additionally, when moving to 72 h of treatment a gene down-regulated by prednisolone was *KIT*, a proto-oncogene homolog to *c-KIT*. Its inhibition has been reported to be involved in leukemia treatment and glucocorticoid activity [86-88]. Further on, a gene that is up-regulated by the high prednisolone dose was *MADD*. This gene is a propagating agent of the death signal induced by TNF. It mediates the signal from TNF to MAPK pathway thus inducing apoptosis. It is reported to be overexpressed in several neoplasms [89] and also it is reported that its over-expression is linked to tumor survival [69].

MADD is a propagating agent of TNF induced death signals, and more specifically, it mediates the signal from TNF to MAPK pathway, thus inducing apoptosis. MADD is phosphorylated by AKT which then inhibits TRAIL-induced apoptosis [69]. Considering the fact that *MADD's *expression pattern is in line with the resistance observed in this study, a lingering question is if this over-expression is the result of an inherent or acquired mechanism.

In the search for shared transcription factor binding motifs an interesting finding was that of JUN. This factor has been reported previously to play a role in the apoptosis regulation in the same system studied here [90].

Chromosome mapping and chromosomal-relative expression did not correlate with the number of genes represented on each chromosome, thus suggesting a well-coordinated GR-induced regulation. GCs are known to lead to chromatin modification about 2 h following treatment. A detectable change in the expression of genes that are immediately regulated by the GC is expected to reach the stage of mRNA steady-state levels at approximately 3-4 h after treatment. Then, genes regulated by the GC, begin to influence subsequent gene expression. cDNA microarray analysis upon 4 h of treatment has been chosen, in order to reduce the complexities that arise later due to ensuing feedback mechanisms, at the same time focusing on the GR/NF-κB-linked, direct target genes [76]. Moreover, the delay in the action of GCs has been reported depending on the cell type and lasting from 2 h to 24 h [91]. Specifically, in our model a prednisolone action lag lasts up to 72 h [76], as reported in different studies [92]. Chromosomal-related expression, is closely linked to chromatin remodeling and it could be the object of future investigations, as it appears that the GR affects almost all chromosomes at all time points as could be easily hypothesized from the abundance of GR response elements throughout the human genome [10]. This confirms the idea of complete genome regulation by the glucocorticoid receptor in cellular systems.

The final remarks regarding resistance to glucocorticoids, comes from pathway analysis. Based on our observations, probably the apoptosis induced by either TNF receptors or NGFB over the AKT pathway and in concordance to the MAPK pathway is ruled out, since AKT remains unchanged. That means that in the present system, glucocorticoids do not significantly affect this pathway. It is also reported that protein-kinase networks control in a major way the observed resistance to glucocorticoids [93]. On the other hand, based on the pathway model, two alternative ways remain; the mitochondria-induced apoptotic pathway and Ca^+2 ^induced pathway. From those two the mitochondria-directed pathway appears to entail genes that remain unchanged from glucocorticoid treatment. If we also take into account the notion that GR translocation to the mitochondria is part of the resistance mechanisms to glucocorticoids [93] then this action could be related to the induction of resistance in our system, as through translocation to mitochondria GR ceases to exert its regulatory effects. Alternatively, a likely mechanism for induction of resistance to glucocorticoid-induced apoptosis could be the Ca^+2 ^signaling pathway. Several key genes in it appear to be differentially expressed, such as *PPP3CC *and *CASP1*, at the early time points, thus making evasion of apoptosis probable.

Attention should be also drawn on two categories of genes regulated by prednisolone. These are metabolic genes and signal-transduction related genes. In both time points, high prednisolone concentration regulates such genes, thus grounding for cell proliferation machinery. In addition, the regulation of NF-κB-related genes implies an inherent mechanism of resistance through the established link of NF-κB inflammatory role and GC-induced resistance.

Overall, the results of the present study support our initial working hypothesis for a rather inherent GC resistance mechanism. However, further exploitation of the proposed computational analysis workflow through the conduct of a larger genome-wide transcriptomic study could promote the extent and level of detail regarding our knowledge about the molecular underpinnings which orchestrate the manifestation and dynamics of the mechanisms of GC resistance and desensitization.

## Conclusions

The leukemic cells used in this study are known to be resistant to glucocorticoids and in particular to prednisolone. In order to gain more insight to the mechanisms of resistance we have developed a rational computational analysis framework along with experimental approaches in order to answer the question of whether cells exhibit this resistance due to an inherent or, an over time, acquired mechanism. We have used the biological information derived from our modeling approach to interpret the findings observed regarding our initial hypothesis. The analysis of the results supports a complex mechanism of action for the cells which tends to favor an intrinsic mechanism of resistance, although in one case prednisolone appeared to exert the expected effects i.e. positive regulation of apoptotic genes, thus supporting an acquired mechanism. It seems that a crucial determinant for the manifestation of either phenotype seems to be the dosage as other haphazard environmental factors, which partly regulate the cellular circuitry towards either direction. The ability to discriminate between acquired or intrinsic mechanisms of resistance is of major importance both in the mechanics of glucocorticoid signal transduction as well as to the clinical praxis since GCs are still front line medications for the treatment of malignant diseases, especially in the case of leukemia.

## List of abbreviations

CGS: common gene set; DE: differentially expressed; GC: glucocorticoid; GO: Gene Ontology; GR: glucocorticoid receptor; NF-κB: nuclear factor kappa beta; SD: standard deviation; SD100: top 100 highest standard deviation genes.

## Competing interests

The authors declare that they have no competing interests.

## Authors' contributions

EGS designed the structure of the preprocessing and analysis pipeline, implemented the corresponding Matlab^® ^scripts, performed the computational analyses and drafted the manuscript. GIL conceived the idea of the hypothetic model of the study, performed the biological experiments, contributed to the biological interpretation of the results and drafted the manuscript. AP supervised the design and implementation of the computational analyses and participated in manuscript revision. DK, SV and FTS contributed in the supervision of various aspects of the work, provided technical consultation and participated in manuscript revision. AAC participated to the conception and design of the study, supervision of the computational analyses, results interpretation and manuscript revision, evaluated the computational analyses results and proofread the manuscript. All authors have read and approved the final manuscript.

## Supplementary Material

Additional file 1 **Cross-platform normalization**. One microarray slide per platform (1.2 k and 4.8 k) was selected and a quantile-quantile plot (QQ-plot) was produced (a) before and (b) after the application of cross-platform normalization. In each QQ-plot, the quantiles of all gene expression values of the first slide were plotted against the quantiles of all gene expression values of the second slide. In the case where the gene expression values of the two slides come from the same distribution, the points in the plot should fall near the straight line. Click here for file

Additional file 2 **Optimal cluster number determination**. K-means clustering was executed for a number of clusters, varying between 2 to 30. For each cluster number, the best (maximum) value of all the average silhouette widths obtained at 1,000 executions was plotted against the cluster number. Since the maximum values of the average silhouette width did not exhibit any specific trend, the optimal cluster number was determined as the one corresponding to the maximum value of the plot, indicated by the arrow. For the computation of the silhouettes the squared Euclidean distance was also used. Click here for file

Additional file 3 **Unified dataset after data integration**. The list of the 490 genes, common in all experimental platforms and replicates (CGS) and their corresponding fold change ratios per experiment. Data integration was performed after (i) matching the reporters on the two microarray platforms through UniGene Cluster IDs, and (ii) applying a cross-platform normalization approach. Click here for file

Additional file 4 **Gene subsets based on the formulations posed for resistance**. The list of genes per subset as derived after intensity-dependent calculation of the standard Z-score, along with the intersections based on the formulations described in Methods. Click here for file

Additional file 5 **GO terms predicted for each gene subset**. The list of the most significant (p < 0.05) GO terms per gene subset, as derived from the GO enrichment analysis. Click here for file

Additional file 6 **Cluster membership of the top 100 genes with the highest standard deviation**. The list of the SD100 gene set, along with the list of genes per cluster showing similar expression profile according to cluster analysis. Click here for file
