# Supplementary figures and images for: Elucidating the identity of resistance mechanisms to prednisolone exposure in acute lymphoblastic leukemia cells through transcriptomic analysis: A computational approach
Source: J Clin Bioinforma. 2011 Dec 20;1:36. doi: 10.1186/2043-9113-1-36 (PMC3313905; doi:10.1186/2043-9113-1-36)

(a)

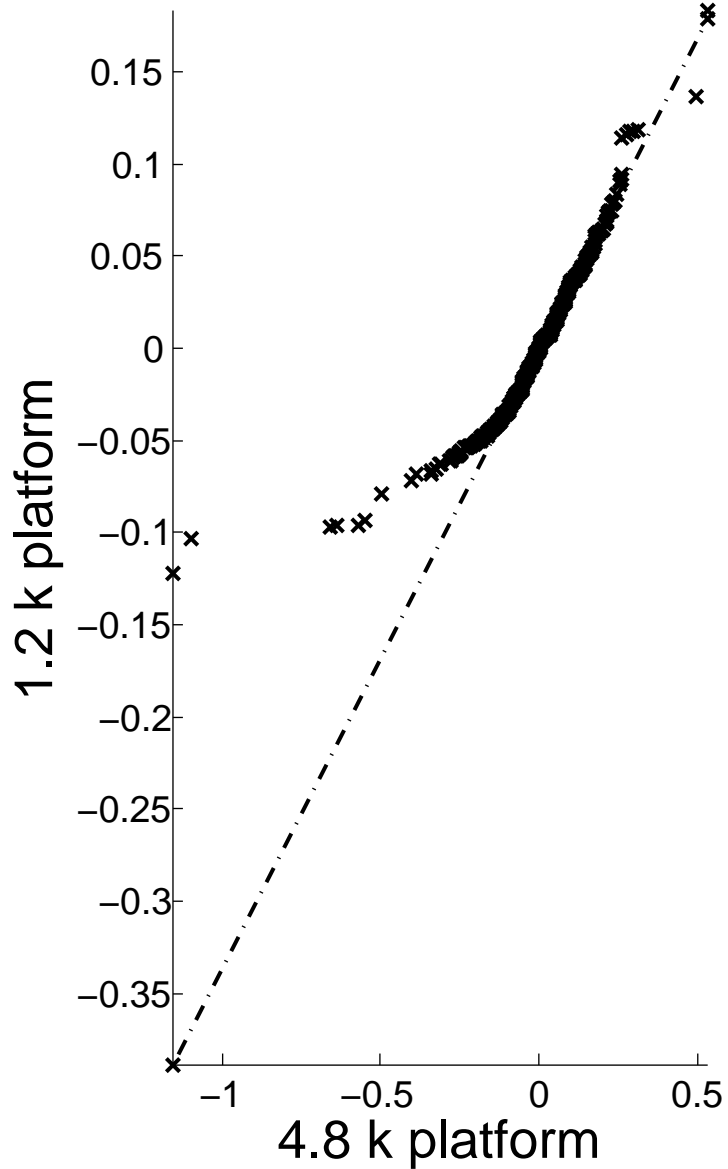

(b)

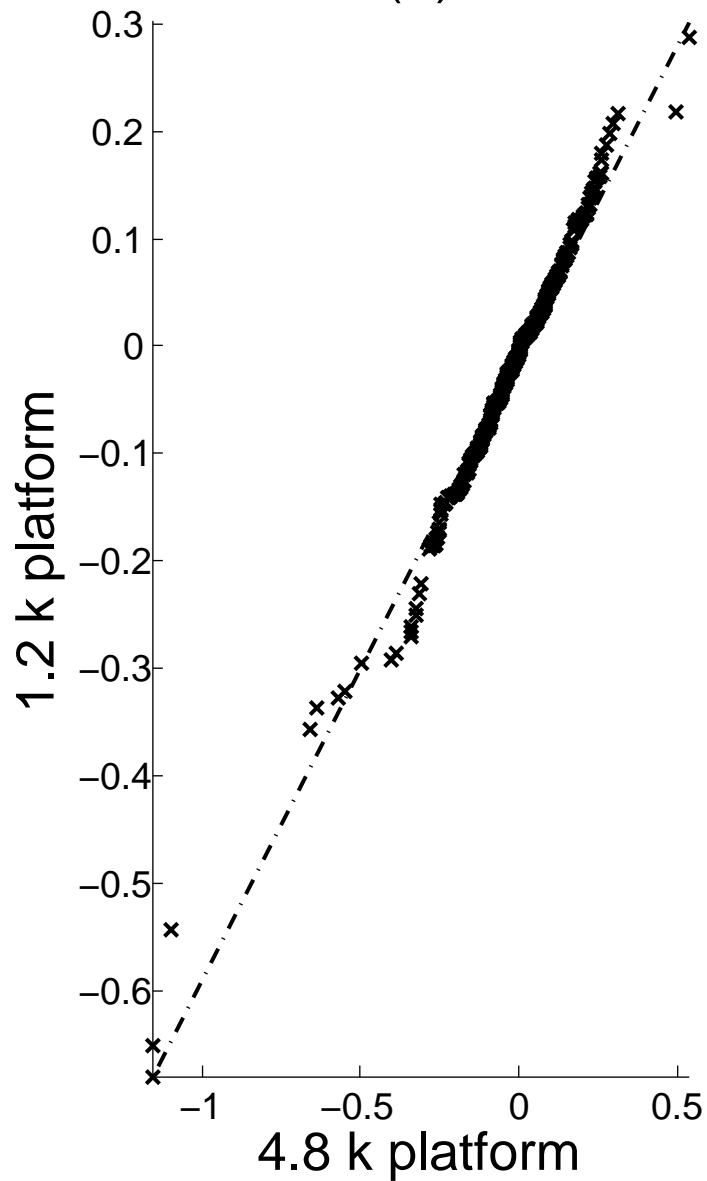

Supplement: Additional file 1 — Cross-platform normalization. One microarray slide per platform (1.2 k and 4.8 k) was selected and a quantile-quantile plot (QQ-plot) was produced (a) before and (b) after the application of cross-platform normalization. In each QQ-plot, the quantiles of all gene expression values of the first slide were plotted against the quantiles of all gene expression values of the second slide. In the case where the gene expression values of the two slides come from the same distribution, the points in the plot should fall near the straight line. [file 2043-9113-1-36-S1.PDF]

Average silhouette width

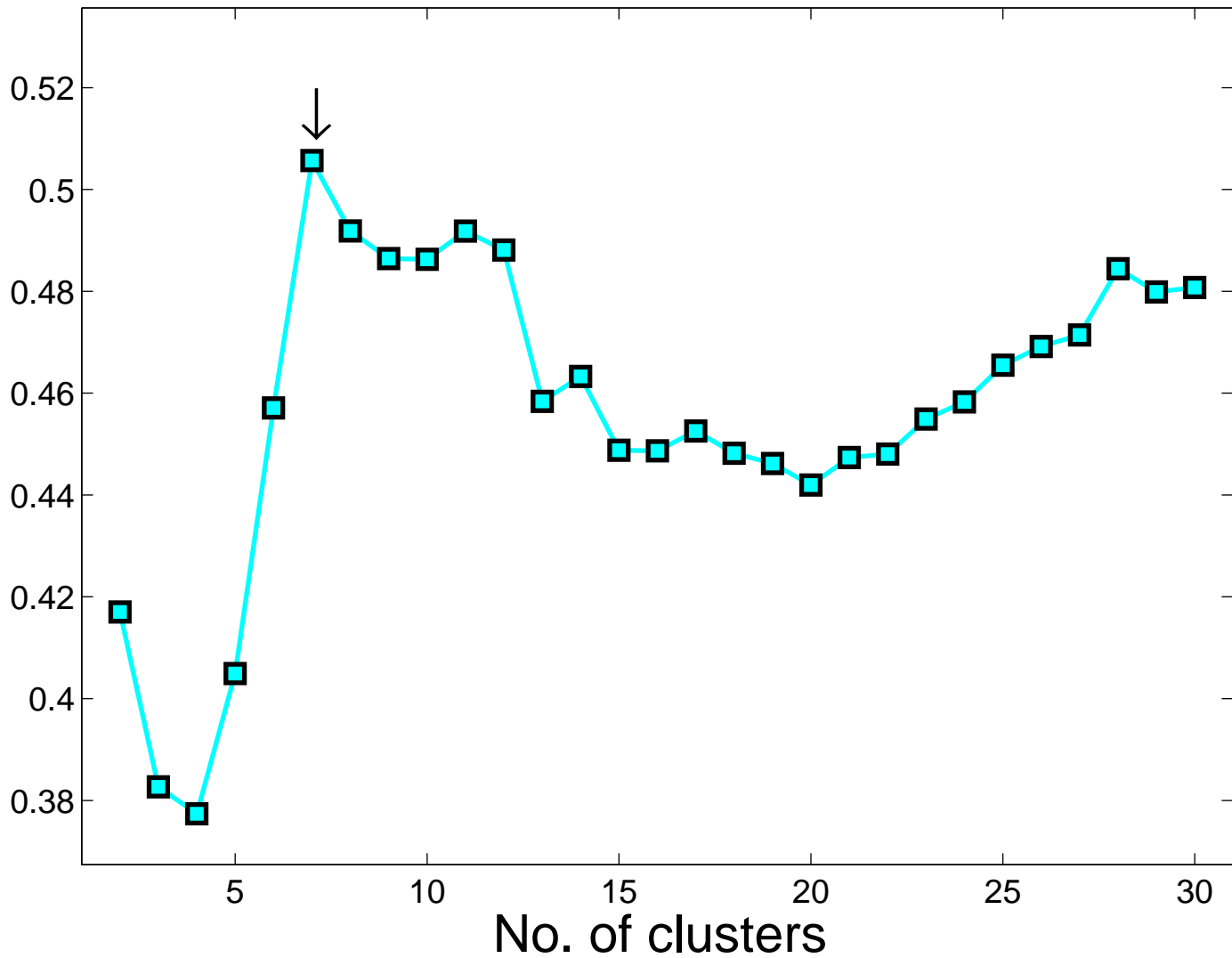

Supplement: Additional file 2 — Optimal cluster number determination. K-means clustering was executed for a number of clusters, varying between 2 to 30. For each cluster number, the best (maximum) value of all the average silhouette widths obtained at 1,000 executions was plotted against the cluster number. Since the maximum values of the average silhouette width did not exhibit any specific trend, the optimal cluster number was determined as the one corresponding to the maximum value of the plot, indicated by the arrow. For the computation of the silhouettes the squared Euclidean distance was also used. [file 2043-9113-1-36-S2.PDF]
